# A decade of *Discovery*: celebrating 10 years of Cell Death Discovery

**DOI:** 10.1038/s41420-025-02779-0

**Published:** 2025-10-20

**Authors:** Ivano Amelio

**Affiliations:** Division of Systems Toxicology, University of Konstanz, Konstanz, Germany

**Keywords:** Cell death, Cell division

Ten years ago, the scientific community was already witnessing an explosive surge in research into the fundamental mechanisms of cell death, apoptosis, necroptosis, pyroptosis, ferroptosis, and emerging forms of programmed cell survival that challenged long-standing dogmas. At the same time, there was a clear need for a platform that could accommodate the rapid publication of interdisciplinary research in cell death biology and related fields across diverse disease contexts, while easing the pressure for novelty and offering the space for reproducibility studies, negative results, and incremental yet important insights, with the sole major requirements of rigor and robustness. In 2015, *Cell Death Discovery* was launched to meet this need, as a sister journal to *Cell Death & Differentiation* and *Cell Death & Disease*.

Today, as we celebrate the journal's 10th anniversary, we can look back at how far we have come, not just as a publication, but as a scientific community connected to, and completing, the CDDpress family. The past decade has been marked by transformative discoveries, the surge of new research fields, and unprecedented appreciation for how cell death interfaces with immunity, infection, cancer, neurodegeneration, and metabolism. Our journal has grown alongside these advances, playing a role in shaping this conversation. *Cell Death Discovery* has provided the perfect venue for studies that may not meet the strict innovation criteria of other journals, yet benefit from rapid processing and timely publication.

Since its inaugural volume in 2015, *Cell Death Discovery* has demonstrated remarkable expansion both in terms of article output and scientific impact. Our bibliometric data show that the published volume grew from 62 articles in 2015, climbing steadily to 483 in 2022, and reaching around 513 in 2024 [1]. The citation metrics also growth impressively providing a stable impact factor range that positions *Cell Death Discovery* in Q1 for the category “Cell Biology” since 2023. With over 13,000 total citations per year, a 2024 impact factor of 7.0, a five-year impact factor of 7.1 [2], and a submission volume exceeding 2200 manuscripts in 2024, these metrics underscore the growing influence of the journal and the trust it has earned within the scientific community. We consider this a proof of success in fulfilling its founding mission of providing an inclusive and efficient platform for the publication of scientifically rigorous research in cell death biology and related areas.

An essential strength of *Cell Death Discovery* lies in its integration within the broader CDD*press* family, which also includes *Cell Death & Differentiation* and *Cell Death & Disease*. Together, these journals form a cohesive ecosystem that has been serving the scientific community for more than 30 years. A pillar of this collaboration is the efficient manuscript transfer system, which allows authors to move their work between journals while retaining reviewer reports and editorial assessments, thereby reducing redundancy, accelerating decision-making, and ensuring that rigorous evaluations are preserved. Close communication among the editorial boards further enhances this process, guaranteeing consistency, fairness, and the highest scientific standards across all three journals. This long-standing trust and reputation make publishing with CDDpress more than a submission, it offers authors the opportunity to engage with a respected, global scientific forum dedicated to advancing cell death research and related fields and its implications for human health.

Research integrity has always been at the core of our mission, especially with the scientific landscape continues to evolve at an unprecedented pace. The rapid development of new experimental techniques, high-throughput technologies, and artificial intelligence tools is transforming the way science is conducted, analyzed, and communicated. While these advances create exciting opportunities for innovation and discovery, they also present challenges in ensuring rigor, reproducibility, transparency, and ethical use. At the same time, the increasingly competitive nature of research careers can fuel toxic environments, where pressures to publish quickly or achieve high-impact results may overshadow the values of good scientific practice. *Cell Death Discovery* also aims to contribute to addressing this issue, providing a platform for sharing negative results, observations, or studies that may not fully meet the requirements from mechanistic insight to biological and clinical relevance. By enabling the dissemination of such work, the journal helps relieve pressure on the community and fosters a balanced publishing venue. *Cell Death Discovery* is committed to the mission to uphold the highest standards of quality, fairness, and editorial integrity, while fostering a culture of respect, accountability, and sustainability in research.

Anniversaries invite reflection, but they are also moments to look ahead. What lies ahead for Cell Death Discovery? We anticipate a continued growing convergence of traditional cell death biology and emerging fields such as single-cell omics, systems biology, and artificial intelligence. The ability to map cell fate decisions at unprecedented resolution will deepen our understanding of how death integrates into tissue homeostasis and disease.

For the journal, our commitment is to remain responsive to these evolving landscapes. We will continue to foster open science, collegial participation, and interdisciplinary conversation. We will invest in highlighting the voices of early-career scientists, fostering inclusivity, and ensuring that the journal reflects the diversity of the community it serves.

Finally, this anniversary is a moment to express gratitude. To our authors, thank you the trusting us with your work. To our reviewers, thank you for the time, expertise, and rigor you bring to every submission. To our editorial board, thank you for your leadership and vision. To our readers, thank you for your engagement, which provide a meaning to our work.
